# A 3D engineered scaffold for hematopoietic progenitor/stem cell co-culture in vitro

**DOI:** 10.1038/s41598-020-68250-5

**Published:** 2020-07-13

**Authors:** Dezhi Zhou, Lidan Chen, Jinju Ding, Xiuxiu Zhang, Zhenguo Nie, Xinda Li, Bin Yang, Tao Xu

**Affiliations:** 1 Biomanufacturing and Rapid Forming Technology Key Laboratory of Beijing, Department of Mechanical Engineering, Tsinghua University, Beijing, People’s Republic of China; 2 Key Laboratory for Advanced Materials Processing Technology, Ministry of Education, Department of Mechanical Engineering, Tsinghua University, Beijing, People’s Republic of China; 3 Plastic Surgery Hospital, Chinese Academy of Medical Sciences and Peking Union Medical College, Beijing, People’s Republic of China; 4 Center for Medical Device Evaluation, National Medical Products Administration, Beijing, People’s Republic of China; 5 Department of Precision Medicine and Healthcare, Tsinghua-Berkeley Shenzhen Institute, Shenzhen, People’s Republic of China; 6 Department of Orthopedics, Fourth Medical Center of PLA General Hospital, Beijing, People’s Republic of China; 7 East China Institute of Digital Medical Engineering, Shangrao, People’s Republic of China

**Keywords:** Biotechnology, Stem cells, Engineering

## Abstract

Proliferation of HPSCs in vitro can promote its broad clinical therapeutic use. For in vitro co-culture, interaction between the stem cell and feeder cell as well as their spatial position are essential. To imitate the natural microenvironment, a 3D engineered scaffold for CD34^+^ cells co-culture was established via 3D bioprinting. Herein, the concentration of hydrogel and the ratio of two kinds of cells were optimized. Flow cytometry, real time PCR and RNA-seq technology were applied to analyze the effect of the engineered scaffold on expanded cells. After 10 days co-culture with the engineered scaffold, the expansion of CD34^+^CD38^−^ cells can reach 33.57-folds and the expansion of CD34^+^CD184^+^ cells can reach 16.66-folds. Result of PCR and RNA-seq indicates that the CD34^+^ cells in 3D group exhibited a tendency of interaction with the engineered scaffold. Compared to 2D co-culture, this customizable 3D engineered scaffold can provide an original and integrated environment for HPSCs growth. Additionally, this scaffold can be modified for different cell co-culture or cell behavior study.

## Introduction

Umbilical cord blood (UC) is considered to be an alternative hematopoietic stem cell source for curing patients with hematologic diseases by allogeneic hematopoietic cell transplantation^1^. However, the limited number of transplantable hematopoietic progenitor or stem cells (HPSCs) is the major obstacle to broad clinical application^2,3^. To date, human HPSCs expansion in vitro is still a challenge because of their easy differentiation during culture^4^. In nature, HPSCs reside in a complex microenvironment, known as niche, containing multiple components of the extracellular matrix (ECM) and various stromal cells including mesenchymal stem cells, adipocytes, osteoblasts, endothelial cells^5,6^. In order to achieve HPSCs expansion in vitro, several strategies, including adding soluble cytokines ^7–9^, culturing with stromal cells ^10,11^ and structuring the 3D environment with different materials ^12–15^ were utilized. All the strategies mentioned above were attempts to mimic the natural conditions for HPSC growth. In recent years, several studies had been processed to prove the superiority of three-dimensional (3D) culture systems in HPSCs proliferation and stemness maintenance ^10,13,14,16–19^. Although these studies made certain achievements, they also had limitations to achieve the natural niche construction due to the multi-cellular composition and complex structure. For instance, in Raic’s study, a porous polyethylene glycol (PEG) hydrogel scaffold was fabricated for 3D co-culture of HPSCs and mesenchymal stem cells (MSCs), but the cells were seeded on the surface of scaffold with a top-down strategy^16^. Moreover, the expansion of stromal cells in the co-culture system may causes the nutrient competition of cells against the expansion of HPSCs. To restrain the growth of stromal cells, methods like mitomycin C treatment ^20^ and irradiation^21^ have been developed for the pre-treatment of feeder cells to restrain their growth. However, the residual mitomycin C from the feeder cells has the potential damage to the HPSCs in the following co-culture. Excessive irradiation can also cause death or mutation of the feeder cells. To solve this problem, a strategy that used hydrogel to encapsulate the MSCs within beads to restrain their proliferation was developed to support HPSCs expansion ^22,23^. But it has limitation to imitate the natural environment because of the absence of structural integrity. According to our group’s previous study, cell bioprinting can deposit the cell-hydrogel, also called bioink, into the preset position to build the desirable 3D structure while the cell viability and function can be well maintained^24,25^. Thus, we managed to establish a 3D structure to imitate for MSCs and HPSCs co-culture via 3D bioprinting.

We got inspiration from the porous structure of bone regions in which HPSCs live^26^. To imitate the interconnect porous structure, we printed the cell-fiber layer in cross to form a grid-like structure, in which the exchange of oxygen and nutrient is maintained efficiently. The millimeter scale was chosen for the consideration of encapsulating the large number of feeder cells. A higher porosity increases the surface area and benefits the exchange of nutrient as well as promote the absorption of growth factors. The scaffold was designed in the millimeter scale with micron-scale hole for HPSCs to host.

**Figure 1:**
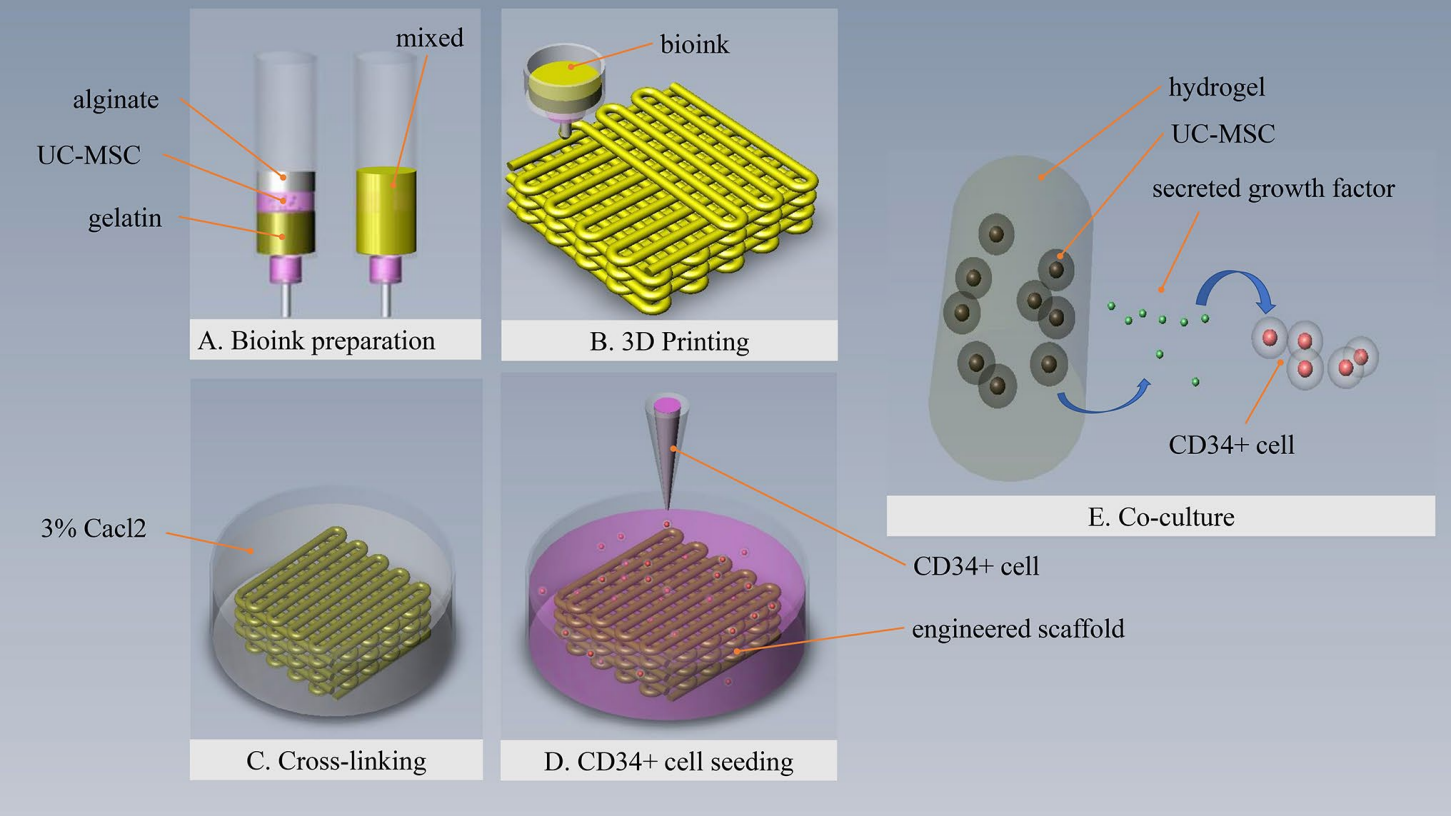
Schematic representation of the preparation, fabrication and function of the engineered scaffold. (**A)** Bioink was composited of medium with UC-MSC, alginate and gelatin and that was mixed uniformly in volume ratio of 1:1:2. (**B**) Bioink was printed to a predesigned structure. (**C**) Cross-linking was through ion calcification between alginate and calcium chloride. (**D**) HPSC medium with CD34^+^ cells was added after washing. (**E**) Functionally maintained UC-MSCs would secrete various growth factors into the medium that were expected to support the expansion of CD34^+^ cell.

Herein, we firstly applied the cell printing technology to construct the cell-laden hydrogel as a 3D structure and subsequently CD34^+^ cells were seeded into the 3D scaffold (Fig. 1A–D). Within this 3D co-culture environment, the feeder cell (UC-MSC) maintain function in the hydrogel and secrete the effective growth factors which passes through the hydrogel to support the CD34^+^ cells culture (Fig. 1E). In this work, the scaffold was optimized and evaluated. Then, the effect of the engineered scaffold on CD34^+^ cells was analyzed and assessed.

## Results

### Preparation and optimization of hydrogel scaffold

**Figure 2:**
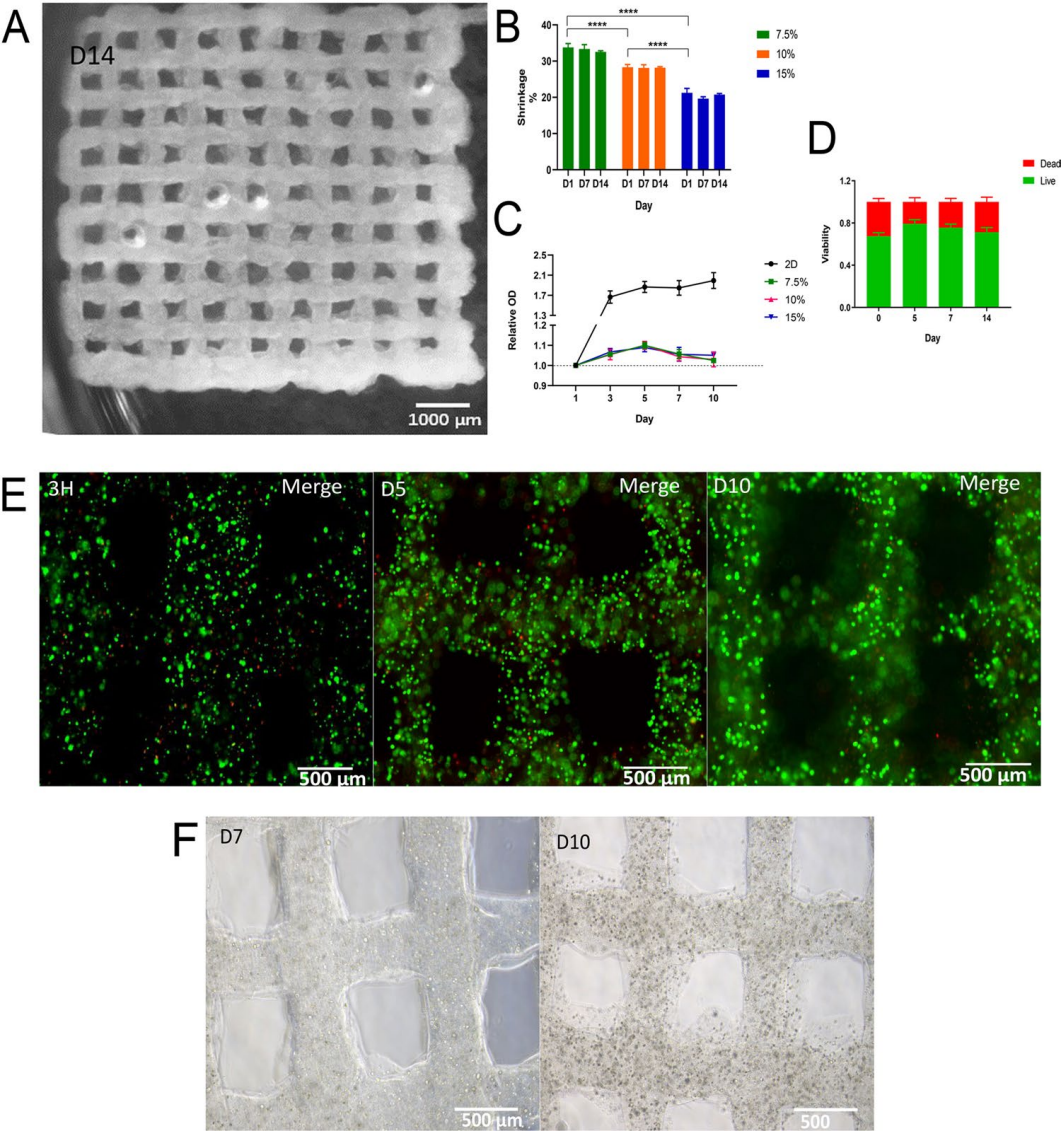
Representative microscopic images of the scaffold with 10% gelatin: (**A**) Image of the scaffold at day 14. (**B**) The shrinkage of scaffolds with different gelatin concentration during the 14 days. The formula: Shrinkage (%) = 100% × measurement/original scale. (**C**) Proliferation rate of UC-MSCs in different culture environments: 2D plate, 3D scaffold with 7.5%, 10% and 15% gelatin. (**D**) The viability of the UC-MSCs in the scaffold with 10% gelatin in 14 days. (**E**) Images of live/dead staining of the scaffold with UC-MSCs encapsulated at day 0, day 5 and day 10 where live cells were green and dead cells were red. (**F**) Images of the scaffold with UC-MSCs encapsulated at day 7 and day 10.

The hydrogel scaffold produced here possessed a porous structure with micron-sized precision as well as a desirable stability that could maintain its shape after 14 days (Fig. 2A). The shrinkage of all groups had no obvious change on the following days (Fig. 2B). According to the OD value of Fig. 2C, during the first five days, UC-MSCs grew quickly in the 2D environment while all UC-MSCs in 3D hydrogel environment maintained a slow upward trend. All groups declined at day 7 but 2D group rose at day 10 while both the 3D group declined little. The data in Fig. 2D showed that the UC-MSCs within 10% gelatin hydrogel can maintain a high survival rate (63–71%) after bioprinting and maintain their viability above 70% during the 14 days culture. To evaluate the leakage of UC-MSCs, the scaffolds were observed twice a day under microscope. Images of the scaffold on various day were taken, and nearly no adherent or suspended cells were found during the culture period (Fig. 2E, F).

Interestingly, we found that the hydrogel scaffold printed following the pre-set size (12 × 12 mm) would shrink after several hours. The scaffolds from all groups shrank and the shrinkage was correlated to their concentrations of gelatin. The scaffold with higher concentration of gelatin showed the less shrinkage (*P* < 0.0001). Moreover, On the other hand, the shrinkage of all groups had no significant change on the following days (D1 vs. D14, *P* = 0.2361 in 7.5% group, *P* > 0.9999 in 10% group, *P* > 0.9999 in 15% group). These results demonstrated that the scaffolds had a long-term stability. Additionally, merging of the layers only appeared in 7.5% gelatin scaffold, but no merging was found in other scaffolds with higher concentration of gelatin, suggesting that the higher concentration of gelatin benefits the structural stability. Compared to the 10% gelatin hydrogel, the 15% gelatin hydrogel showed poor performance in cell viability maintaining after bioprinting and that may raise concern for the hardness of the hydrogel. In consideration of the accompanying hardness from the higher concentration of gelatin, the 10% gelatin hydrogel was chosen for the following experiments to maintain a higher level of viability of UC-MSCs. From curve of 2D group (Fig. 2C), we concluded that due to the limitation of the plate surface area and the nutrient, UC-MSCs would slow down their growth rate and some unhealthy cells detached from the plate, but they grew again with fresh medium supplement as well as new space. In this way, the physiological circulation of 2D culture would consume lots of nutrients in the co-culture system and detached feeder cells would be included when the expanded cells of CD34^+^ cells were collected.

For the proliferation of UC-MSCs in 3D scaffold, no significant differences among the 7.5%, 10% and 15% were found (Fig. 2C) (*P* values range from 0.3515 to 0.9999). The 10% gelatin scaffold was taken as the example for further analysis. The cell number of MSCs in the gelatin scaffold increased dramatically over the first 5 days (Day 1 vs. Day 5, *P* = 0.0009), and then decreased gradually but not significantly (Day 7 vs. day 5, *P* = 0.2321; Day 10 vs. Day 7, *P* = 0.9999). On day 7 the cell number did not decrease and maintained a high value for the rest of the culture period. One possible reason is that the cells would not grow much but preserve the viability. This phenomenon is correlated well with the other report ^22^ in which the number of MSCs increased at first then declined gradually, suggesting that the material of alginate used in this study is biocompatible but not facilitated the MSCs growth. Moreover, through the culture, we monitored the leakage of the MSCs via observing the scaffold under microscope and nearly no cells were escaped during the culture time.

### The effect of CD34^+^ cell medium on UC-MSC

**Figure 3:**
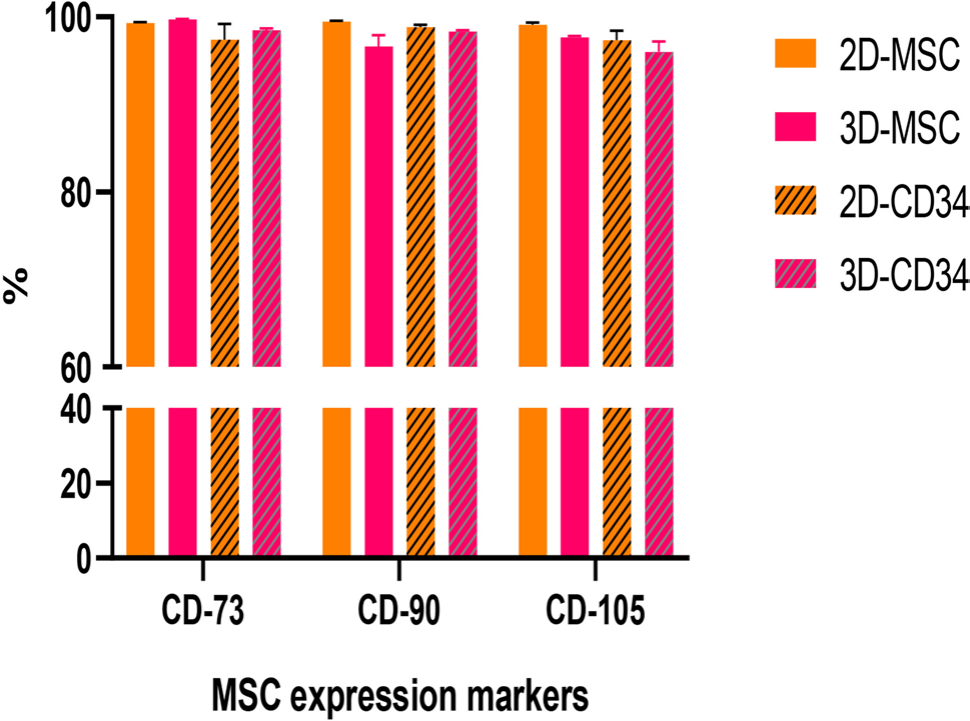
Expression marker of 10 day-expanded UC-MSCs in different culture medium group.

CD34^+^ cell medium may induce MSCs’ differentiation. The influence of it on UC-MSCs should be considered. To evaluate the influence of CD34^+^ cell medium on MSC, we used flow cytometry to assess their differentiation after 10 days culture. Three key markers (CD73, CD90, CD105) of MSCs were evaluated. Both MSCs cultured in 2D or 3D environment with CD34^+^ cell medium exhibit a high expression of three markers (above 90%, Fig. 3), and the result showed that no MSC differentiation appeared in HPSC culture medium.

### Optimization of seeding density ratio of CD34^+^ cells to UC-MSCs for co-culture

**Figure 4:**
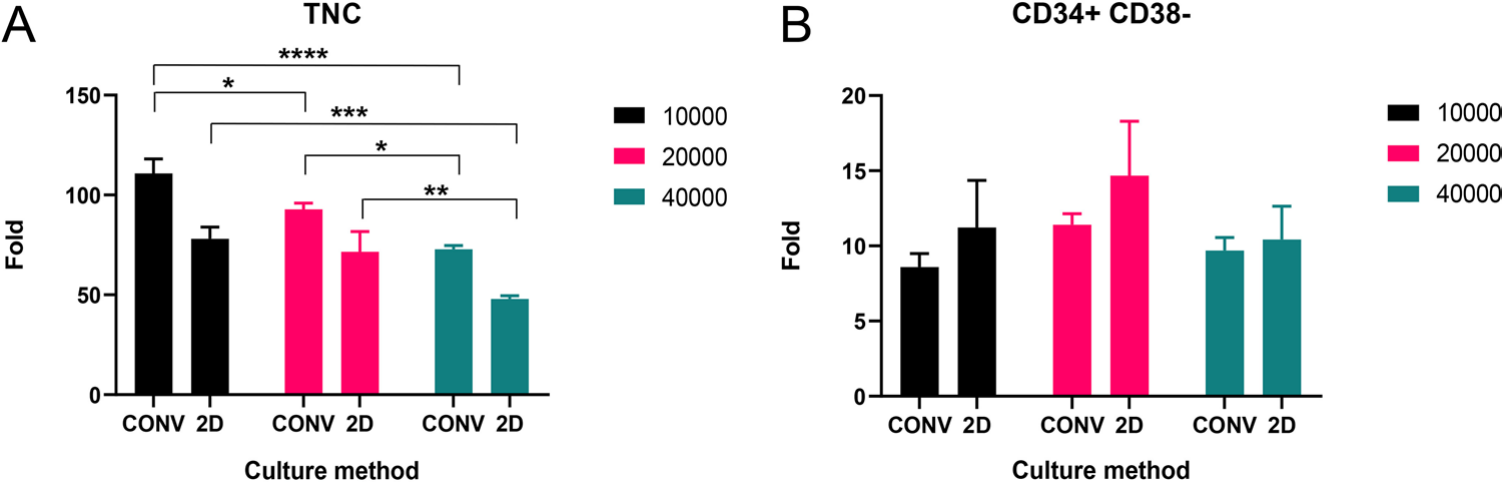
The expanded fold of the TNCs (**A**) and the CD34^+^CD38^−^ cells (**B**) from different initial CD34^+^ cells number at day 10.

A proper ratio of CD34^+^ cells to UC-MSCs can enhance the proliferation of CD34^+^ cells. To define the proper ratio, co-cultures of the CD34^+^ cells with different number of 10,000, 20,000 and 40,000 to 2 × 10^5^ UC-MSCs were performed respectively. Compared to other groups, TNCs from 10,000 CD34^+^ cells had the largest expanded fold while the expanded fold of TNCs from 40,000 CD34^+^ cells was lowest (Fig. 4A). Expanded fold of CD34^+^CD38^−^ cells was highlighted in 20,000 CD34^+^ cells group (Fig. 4B).

In the co-culture of CD34^+^ cells with UC-MSCs, nutrient competition and waste substance caused by the UC-MSCs may regulate CD34^+^ cells. In Pan’s work^22^ and Fujimoto’s work^23^ , the results exhibited that existence of excess feeder cells decreased the expansion of CD34^+^ cells, which was related to harmful metabolites produced by the excess number of feeder cells. Similarly, our result showed that a high initial number of CD34^+^ cells (40,000) decreased the expanded fold of cell and it may be caused by the inevitable competition of bioactive factors and nutrients in co-culture system. Furthermore, the inhibitory factors accumulation produced by the expanded cells may also limits the growth of CD34^+^ cells^27^.

As shown in Fig. 4, CD34^+^ cells with lower initial number may be beneficial to TNCs proliferation. However, the expanded fold of CD34^+^ cells was highlighted in 20,000 CD34^+^ cell group. These results suggested that, the growth of CD34^+^ cells was related the ratio of them. To maximize the supporting function of UC-MSCs, CD34^+^ cells seeded at a seeding density ratio of 1: 10 to UC-MSCs was employed for further experiments.

### Effect of hydrogel on CD34^+^ cell

**Figure 5:**
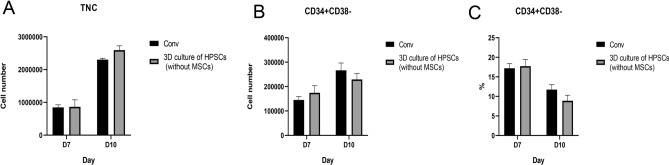
Quantification of TNCs (**A**) and CD34^+^ CD38^−^ cells (**B**) in conv and 3D culture of HPSCs group. (**C**) Expression of CD34^+^CD38^−^ phenotype in conv and 3D culture of HPSCs group.

3D culture of HPSCs without UC-MSCs was set as experimental group and the culture plate without UC-MSCs was set as conventional group (conv). 20,000 CD34^+^ cells were seeded in both groups, respectively. As shown in Fig. 5A, no significant difference in the number of TNCs between the conv and 3D groups. According Fig. 5B, C, expression of CD34^+^CD38^−^ had no difference between conv and 3D groups. These results indicated that the hydrogel scaffold without MSCs had nearly no effect on CD34^+^ cells.

### Effect of 3D engineered scaffold on CD34^+^ cell

**Figure 6:**
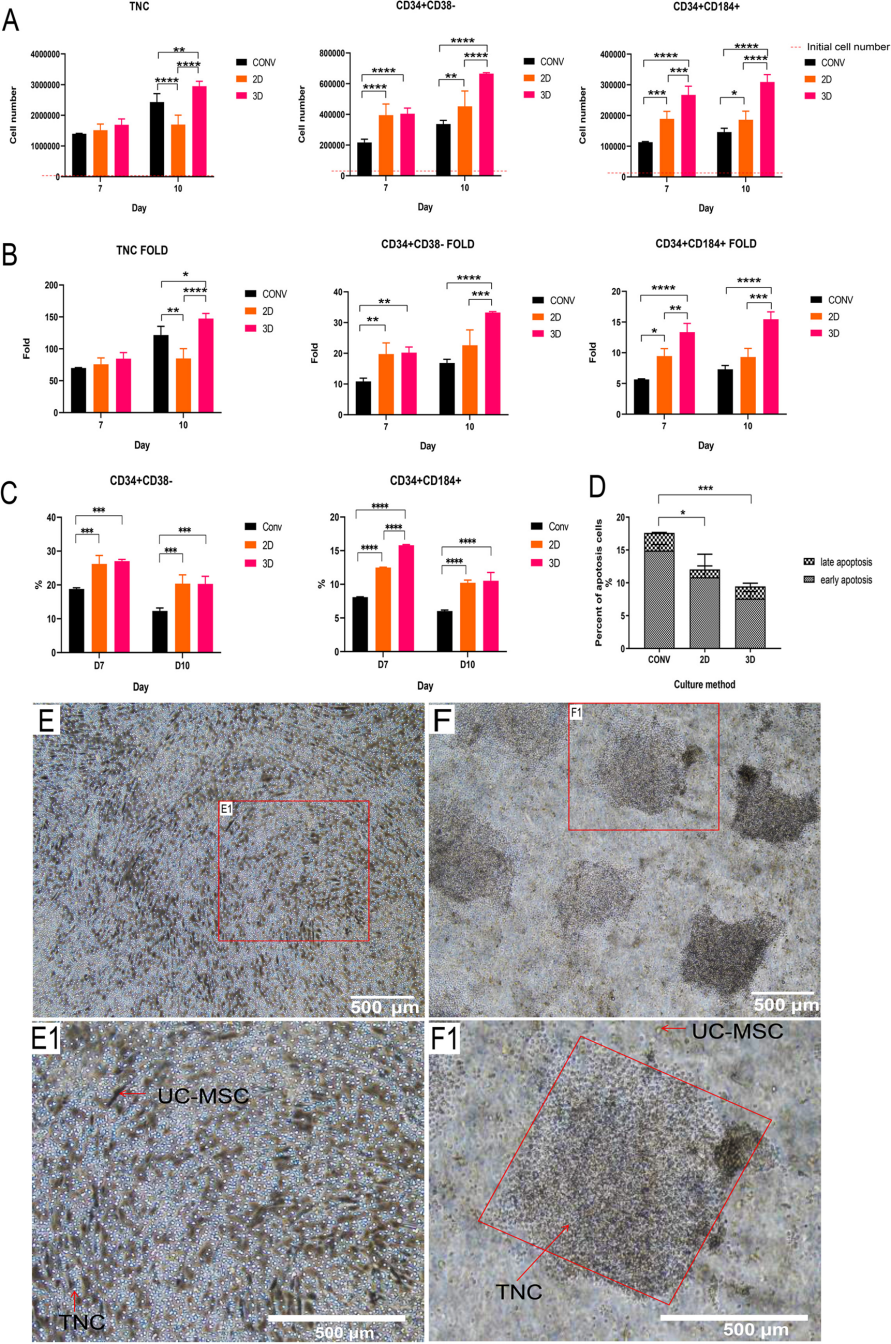
Quantification of harvest cells in different groups at day 7 and day 10: (**A**) cell number, (**B**) expanded fold. (**C**) Expression of CD34^+^CD38^−^ and CD34^+^CD184^+^ phenotype in different groups. (**D**) Quantification of apoptosis of harvest cells in different groups at day 10. (**E** & **F)** Representative images of CD34^+^ cells co-culture with UC-MSCs at day 10. Optical microscopy images: (**E**&**E1**) Co-culture in 2D environment. (**F**&**F1**) Co-culture in 3D environment.

Data in Fig. 6A indicated that number of cells (TNCs, CD34^+^CD38^−^, CD34^+^CD184^+^) in 2D group had no obvious increase while the number of cells in 3D group increased dramatically during day 7–10. Both 2D and 3D groups had a higher expanded fold than conv groups (Fig. 6B). Compared to the 2D group (27.61-folds) the expansion of CD34^+^CD38^−^ cells in 3D group can reach 33.57-folds. Additionally, the expanded fold of CD34^+^CD184^+^ cells in the 3D group was 16.66-folds which was higher than that of the 2D group (10.72-folds) (Table 1). The expression of CD34^+^CD184^+^ between 2D and 3D group had difference at day 7, but no obvious differences on expression of CD34^+^CD38^−^ or CD34^+^CD184^+^ were found at day 10(Fig. 6C). The density plots of flow cytometry were in shown in supplement Fig. 1. The apoptosis of expanded cells from CD34^+^ cells was also evaluated. The conv group exhibits a higher proportion of early apoptosis and late apoptosis (Fig. 6D). Compared to the dispersive cells in 2D group, the suspended cells can aggregate inside the hole of 3D engineered scaffold (Fig. 6E, F). In 2D co-culture environment, the suspended cells with circular shape (10–12 μm) were TNCs while the adherent cells (attached to the plate) with spindle shape were UC-MSCs (Fig. 6E1). In 3D co-culture environment, the TNCs aggregated and hosted in the hole of the scaffold while the UC-MSCs were encapsulated within the hydrogel scaffold (Fig. 6F1).

**Table 1:** Fold of cell expansion at day 10.

	Con	2D	3D
TNC	121.54 ± 13.76	84.94 ± 15.3	147.44 ± 8.03
CD34^+^CD38^−^	16.85 ± 1.2	22.61 ± 5	33.29 ± 0.28
CD34^+^CD184^+^	7.31 ± 0.63	9.31 ± 1.41	15.46 ± 1.2

CD184(CXCR4) is one essential marker of CD34^+^ cells with expression of homing^12,28^. The higher portion of CD34^+^CD184^+^ cells in 3D group indicated that the scaffold may had better performance in mimicking the microenvironment compared to the monolayer feeder cells.

There is no significant difference in TNCs expansion among the conv, 2D and 3D groups on day 7. This phenomenon may highly relate to the sufficient nutrients in the medium during the early culture. But with the growth of the cells, the medium may be no longer sufficient for the subsequent culture. In this situation, the feeder cells competed with the target cells for the nutrient in the medium. Thus, the expansion of TNCs in 2D co-culture had almost no increase on day 10 (Fig. 6A). While the growth of UC-MSCs in 3D co-culture environment was restrained by scaffold. Therefore, more nutrient could be supplied for the proliferation of CD34^+^ cells. Based on these, we conclude that the 3D engineered scaffold enhanced the proliferation of CD34^+^ cell compared to the 2D co-culture environment.

Apoptosis is essential for regulating the number of cells during the growth of CD34^+^ cells in vitro. To evaluate the apoptosis and the vitality of TNCs from different conditions, TNCs after 10 days culture in vitro were analyzed. As shown in Fig. 6D, the TNCs culture without UC-MSCs exhibits a higher proportion of early apoptosis but a similar proportion of late apoptosis as the co-culture group. However, there is no significant difference between the 2D and 3D group both in early and late apoptosis. The TNCs from the both 2D and 3D co-culture environment exhibited better cell vitality than conv group, suggesting that UC-MSCs may reduce the early apoptosis of CD34^+^ cells.

### Biological environment of the engineered scaffold

**Figure 7:**
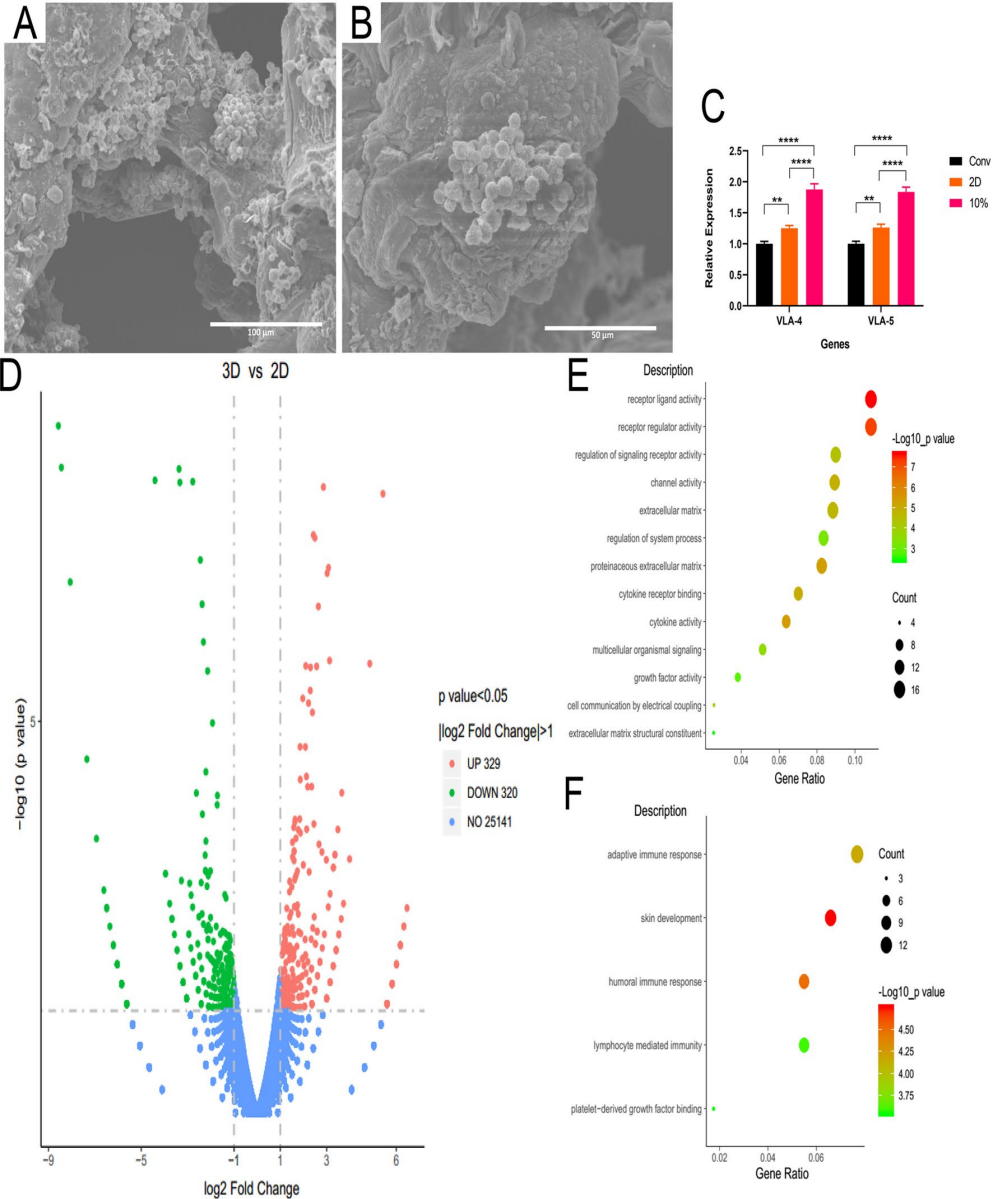
SEM images of engineered scaffold. Respective scale bars: (**A**) 100 μm; (**B**) 50 μm. (**C**) Relative fold increase homing genes in 2D and 3D culture systems after 10 days culture. Data are representative of 2 independent experiments with similar results. (**D**) Volcano plot of differential expression between TNCs in 2D and 3D group. 2D was set as the control group. Data are representative of 2 independent experiments with similar results. GO enrichment analysis of differentially expressed genes exhibit statistically enriched GO categories for upregulated (**E**) and downregulated (**F**) genes. Data are representative of 2 independent experiments with similar results.

SEM images of scaffold were taken after co-culture to assess the interaction between TNCs and the scaffold. As shown in Fig. 7A, B, the suspended cells grew as cluster on the surface of the scaffold which meant that the hydrogel scaffold may enhance their behavior such as aggregating. The expression of VLA-4 and VLA-5 were evaluated via real time PCR. The expression of VLA-4 and VLA-5 in 3D group were obviously higher than that in conv and 2D group (Fig. 7C). For deeper insight into the behavior of CD34^+^ cells between 2D and 3D environment, we conducted expression profiling employing RNA-seq. As presented in Fig. 7D, 329 genes were found to be significantly upregulated and 320 genes were found to be significantly downregulated while expression of more than 20,000 genes showed no significant change in 3D populations compared to that in 2D group. Then gene ontology (GO) enrichment analysis of the biological process terms for both the up- and down-regulated gene groups were carried out. Specifically, we focus on the terms which are associated with cell–cell and cell–environment interaction, that were statistically enriched in the upregulated gene set (Fig. 7E). Additionally, we found terms that were statistically enriched in the downregulated gene set are inclined to the immunity (Fig. 7F).

VLA-4 and VLA-5 regulate the adherent ability of HPSCs to ECM^29^. They are required at high levels for efficient homing of circulating HPSCs into the bone marrow niche^5,6,29^. From our data in Fig. 7C, compared to the 2D co-culture, the gene expression of both VLA-4 and VLA-5 in 3D co-culture group was upregulated (*P* < 0.0001), suggesting that the 3D co-culture environment may be closer to the natural environment for HPSC growing. At the same time nearly no cells aggregation was seen in 2D co-culture environment, so that the 3D co-culture might enhance most of the cell behaviors including the cells aggregating. Therefore, the higher expression of VLA-4 and VLA-5 may relate to the cluster of TNCs on the surface of the scaffold. In 3D-coculture environment, cells were more likely attached to the scaffold and proliferated, suggesting that CD34^+^ cells in 3D co-culture group had more potential ability to expand^14^. Furthermore, the GO enrichment analysis showed that the behaviors of cells in 3D co-culture environment were enhanced compared to the cells in 2D co-culture environment.

## Discussion

The alginate-gelatin hydrogel scaffold printed via 3D bioprinting can be applied for long-term co-culture due to their biocompatibility and stability. The hydroscopicity of porous hydrogel benefits the exchange of the nutrients and oxygen in 3D structure in which the UC-MSCs can obtain enough supplement to maintain a high viability, and the secreta of the cells can be released. The suppression of UC-MSCs proliferation reduced the effect competition of nutrients in 3D co-culture system so that the TNCs had less apoptosis, higher viability as well as higher expanded fold. This suppression ability of the engineered scaffold may be applied in co-culture for cell proliferation. The increasing expression of VLA-4 and VLA-5 in 3D engineered scaffold demonstrated that the cells in 3D co-culture environment had a higher potential of proliferation. Furthermore, the upregulated genes on GO enrichment analysis indicated that the 3D co-culture environment may be closer to the natural environment for HPSC growing. These results suggested that the 3D engineered scaffold may provide an original and integrated environment for HPSC growth compared to 2D co-culture environment. And our work demonstrated the feasibility of 3D bioprinting in structuring the co-culture environment for HPSCs and UC-MSCs.

The UC-MSCs helped maintain the stemness of CD34^+^ cells and promoted their proliferation in both 2D and 3D co-culture environment. Compared to 2D co-culture environment, the number of CD34^+^CD38^−^ and CD34^+^CD184^+^ cells was highlighted. However, the phenotype of CD34^+^ has no obvious difference between the 2D and 3D groups. It may relate to the hydrogel. Materials play an important part in regulating HPSCs in vitro. Natural materials such as fibrin, collagen and fibronectin were proved to enhance the proliferation of HPSCs^14,28,30,31^. The superiority of special synthetic materials in stemness maintenance of HPSCs was also reported^15,32^. Thus, new materials development for engineered scaffold would be the next key point. And the engineered scaffold for co-culture will be optimized for biomedical researches such as cell interaction, cells migration in the future.

## Conclusion

3D bioprinting can be applied in engineered microenvironment construction for cell culture. In this work, we provided a 3D engineered scaffold for CD34^+^cells and UC-MSCs co-culture. And the results demonstrated its superiority in biomimetic culture compared to the 2D culture system. Additionally, this scaffold exhibited potential application for cell–cell and cell–matrix study.

## Methods

### UC-MSC culture

UC-MSCs were purchased from SCIENCELL (Carlsbad, CA). UC-MSCs were maintained in a cultivation medium consisting of human MSC basal medium (STEMCELL, Vancouver, Canada), supplemented with human MSC stimulatory supplement (STEMCELL, Vancouver, Canada) and 1% penicillin–streptomycin. Cells were seeded in 75 cm^2^ flask with 10 mL of medium for expansion. Medium was completely changed every 3 days and cells were harvested at a confluence of 80–85%. The passage of UC-MSC utilized in the experiment was 3 to 6.

### CD34^+^ cell culture

Frozen human umbilical cord (UC) CD34^+^ cells (> 90% CD34^+^, > 95% viability) were purchased from NOVOBIOTECHNOLOGY (Beijing, China). CD34^+^ cells culture was performed using STEMSPAN SFEM II medium (STEMCELL, Vancouver, Canada) supplemented with 1% penicillin and streptomycin and 50 ng/mL recombinant human stem cell factor (PROTEINTECH, Chicago, USA), 50 ng/mL recombinant human thrombopoietin (PROTEINTECH, Chicago, USA) and 50 ng/mL recombinant human Flt3-Ligand (PROTEINTECH, Chicago, USA). All culture experiment are under the condition of humidified air with 5% CO_2_ in 37℃.

### Bioink preparation

Gelatin and sodium alginate were purchased from ALADDIN (Shanghai, China). Sodium alginate and gelatin were dissolved respectively in 0.9% sodium chloride solution (w/v). 4% (w/v) alginate was unaltered and different concentrations of gelation (7.5%, 10%, 15%, w/v) was chosen to experiment, respectively. Before the experiment, these solutions were sterilized by both pasteurization and UV sterilization. 3% calcium chloride solution (w/v, CaCl_2_) was sterilized for crosslinking. For bioprinting, UC-MSCs were isolated and resuspended in the MSC medium. Then, cell suspension, sodium alginate solution and gelatin solution at various concentrations were mixed at a volumetric ratio of 1:1:2 to produce 1 mL bioink with a final concentration of 2 × 10^6^ mL UC-MSCs (Fig. 1A).

### Preparation and optimization of hydrogel scaffold

Scaffold was printed by Livprint 3D bio-printer NORM series (MEDPRIN, China). To mimic the natural porous structure of bone regions, a square grid scaffolds were designed with the size of 12 × 12 mm cross sectional area and 2.1 mm thickness. Optimal printing parameters were chosen. Briefly, the printing chamber temperature was set at 8℃, 0.26 mm diameter nozzle was selected, and the scanning speed was controlled at 4 mm/s (Fig. 1B). After printing, cell-laden hydrogel scaffolds were immersed in 3% (w/v) CaCl_2_ solution for 3 min for cross-linking the sodium alginate (Fig. 1C). All scaffolds were gently washed with phosphate buffer solution (PBS) twice to remove the CaCl_2_ solution.

### Preparation of co-culture system

As mentioned above, UC-MSCs were added into the medium to prepare the bioink. Then, each printing niche consumes approximately 0.1 mL bioink and the number of cells in each niche was about 2 × 10^5^. After washing and transferring the niches into the 24-well culture plates, 2 mL fresh MSC medium was added to each well, and these niches were cultured for 24 h. After 24 h, the MSC medium was removed and 2 ml fresh CD34^+^ cell medium was added after washing with PBS. Subsequently, CD34^+^ cells were added to each scaffold (Fig. 1D). Medium was completely changed every 3 days.

### Scanning electron microscopy (SEM) analysis

The engineered scaffolds after 10 days culture were fixed overnight at 4℃ with 4% glutaraldehyde, and dehydrated by soaking the samples in each gradient ethanol solutions (70%, 80%, 90%, 95%, 100%, and 100%) for 30 min. Following this, the samples were dried under vacuum freeze–drying. Simples were treated by spray-gold and images of the surfaces were photographed by Quanta 200 Scanning Electron Microscope (FEI, Netherlands).

### Cell proliferation assay

For UC-MSCs, Alamar Blue Kit (YEASEN, Shanghai, China) was used to evaluate cell proliferation according to manufacturer’s instruction. Alamar Blue is an indicator of redox, which can be used for quantitative analysis of cell activity and cell proliferation and in vitro cytotoxicity study. For proliferation experiments, the initial cell number of both 2D and 3D samples was 2 × 10^5^. The optical density (OD) value of supernatant was read on a microplate reader (Epoch 2, BIOTEK, USA) at wavelengths of 570 and 600 nm. All groups of OD values were normalized to day 1 for plotting and statistics. For HPSCs, proliferation of total nucleated cells (TNCs) was assessed by cell counter (Countstar Rigel S2, COUNTSTAR, China) and the number of CD34^+^ cells was calculated according to the result of flow cytometry analysis.

### Cell viability

Cell viability was assessed by fluorescent live/dead assay kit (KEYGEN BIOTECH, Nanjing, China) following the protocol. Briefly, medium was removed then the scaffold was washed three times with PBS. Subsequently, PBS solution mixed with 2 μM Calcein-AM and 8 μM propidium iodide was added to stain hydrogel scaffolds. Scaffolds were immersed in the staining solution at room temperature in the dark for 10 mins, then washed three times with PBS. Images were obtained from fluorescence microscopy (Eclipse Ti2-U, NIKON, Japan). The live and dead cells of each sample were counted in six random fields at 100× magnification.

### Cell harvest

As suspension-cultured cells, TNCs were harvested from the scaffold by gently pipetting. After collecting the culture supernatant, PBS was added to wash for three times and subsequently the PBS was collected. TNCs were collected via centrifugation. To dissolve the hydrogel scaffold, 55 mM sodium citrate (Sigma-Aldrich) and 20 mM EDTA (Sigma-Aldrich) in 0.9% NaCl solution was prepared to de-crosslink of alginate^33^. The scaffold was dissolved under mild conditions, the MSCs were recycled via centrifugation, and subsequently the MSCs were reseeded in the culture plate to remove debris. After 6–8 h, MSCs were detached and collected for further analyses.

### Flow cytometry analysis

The phenotype maintenance of CD34^+^ cells and UC-MSCs was analyzed by flow cytometry. Monoclonal antibody CD34-PE (BD Pharmingen, San Diego, CA), CD38-APC (BIOLEGEND, San Diego, CA) and CD184-APC (BIOLEGEND) were utilized for CD34^+^ cells analyses. Monoclonal antibody CD73-PE (eBioscience, Vienna, Austria), CD90-FITC (eBioscience) and CD105-PE (eBioscience) were utilized for UC-MSCs analyses. After adding monoclonal antibody, the cells were incubated in dark at room temperature for 20 min. Cells were washed with PBS with 2% FBS and resuspended for testing. Control samples without staining were included to confirm specificity and for compensation settings. At least 10,000 events were acquired on the flow cytometer (CytoFLEX, Beckman Coulter, USA). Data were analyzed on the CytExpert.

### Apoptosis assay

Apoptotic cell death of the harvested cells under each set of culture conditions was analyzed at day 10, respectively. The cells were stained by Annexin V-FITC (BEYOTIME, Shanghai, China). Following the manufacturer’s protocol, the samples were analyzed by flow cytometry respectively. The percentage of viable cells, cells in early apoptosis and cells in late apoptosis were determined.

### Real time PCR

Expanded cells cultured in different environments were collected and then centrifuged. After centrifugation, cells were completely dissociated with Trizol (Invitrogen, Carlsbad, CA), and total RNA was extracted according to the manufacturer’s protocol. Reverse transcription was performed using ImProm-IITM Reverse Transcription System (Promega, A3800). DNA transcription was carried out using SYBR Green qPCR Super Mix (Invitrogen, Carlsbad, CA). Thermo cycling was performed using ABI PRISM 7,500 Sequence. The data are presented as the relative expression of the genes of interest relative to the internal control gene as determined by the 2^−ΔΔCt^ method. The primers used for real-time PCR for all gene amplifications are shown in Table 2^28^.

**Table 2:** Primer sequences used for real-time PCR.

Gene of interest	Forward primer	Reverse primer
VLA-4	ATGTTGCGCATGTTCTACTG	AGCCTTCCACATAACATATGAG
VLA-5	CAGATCCTCAGCAAGAATCTC	CGTAACTCTGGTCACATATAGG
GAPDH	TGCACCACCAACTGCTTAGC	GGCATGGACTGTGGTCATGAG

### RNA-seq expression analysis^15^

After extraction, RNA degradation and contamination were monitored on 1% agarose gels. RNA purity was checked using the NanoPhotometer spectrophotometer (IMPLEN, CA, USA). RNA concentration was measured using Qubit RNA Assay Kit in Qubit2.0 Flurometer (Life Technologies, CA, USA). RNA integrity was assessed using the RNA Nano 6000 Assay Kit of the Bioanalyzer 2,100 system (Agilent Technologies, CA, USA). Sequencing libraries were generated using NEBNext UltraTM RNA Library Prep Kit for Illumina (NEB, USA) following manufacturer’s recommendations and index codes were added to attribute sequences to each sample. Then library quality was assessed on the Agilent Bioanalyzer 2,100 system. The clustering of the index-coded samples was performed on a cBot Cluster Generation System using TruSeq PE Cluster Kit v3-cBot-HS (ILLUMIA, USA) according to the manufacturer’s instructions. After cluster generation, the library preparations were sequenced on an Illumina Hiseq platform and 125 bp/150 bp paired-end reads were generated.

### Statistical analysis

All data are presented as mean ± SD. Statistical significance was evaluated by analysis of variance using GraphPad Prism 7.0. Differences were considered to be significant for *P* < 0.05. *P < 0.05; **P < 0.01; ***P < 0.001; ****P < 0.0001.Each experiment was performed in triplicate (n = 3) on at least independent three samples (N ≥ 3).

## Supplementary information

Supplementary figure 1
