# Supplementary material for: A 3D engineered scaffold for hematopoietic progenitor/stem cell co-culture in vitro
Source: Sci Rep. 2020 Jul 13;10:11485. doi: 10.1038/s41598-020-68250-5 (PMC7359311; doi:10.1038/s41598-020-68250-5)
Supplement: Supplementary file 1 — Supplementary figure 1 [file 41598_2020_68250_MOESM1_ESM.docx]

**A 3D engineered scaffold for hematopoietic progenitor /stem cell co-culture in vitro**

Dezhi Zhou^1,2^, Lidan Chen^3^, Jinju Ding^4^, Xiuxiu Zhang^5^, Zhenguo Nie^6,7^, Xinda Li^1,2^, Bin Yang^3, *^, Tao, xu^1,2,5, *^

1. Biomanufacturing and Rapid Forming Technology Key Laboratory of Beijing, Department of Mechanical Engineering, Tsinghua University, Beijing, People’s Republic of China

2. Key Laboratory for Advanced Materials Processing Technology, Ministry of Education, Department of Mechanical Engineering, Tsinghua University, Beijing, People’s Republic of China

3. Plastic Surgery Hospital, Chinese Academy of Medical Sciences and Peking Union Medical College, Beijing, People’s Republic of China

4. Center for Medical Device Evaluation, National Medical Products Administration, Beijing, People’s Republic of China

5. Department of Precision Medicine and Healthcare, Tsinghua-Berkeley Shenzhen Institute,

Shenzhen, People’s Republic of China

6. Department of Orthopedics, Fourth medical center of PLA general hospital, Beijing, People’s Republic of China

7. East China Institute of Digital Medical Engineering, Shangrao, People’s Republic of China

# Corresponding author: Tao Xu: [taoxu@mail.tsinghua.edu.cn](mailto:taoxu@mail.tsinghua.edu.cn), Bin Yang: [ybdoctor_psh@163.com](mailto:ybdoctor_psh@163.com)


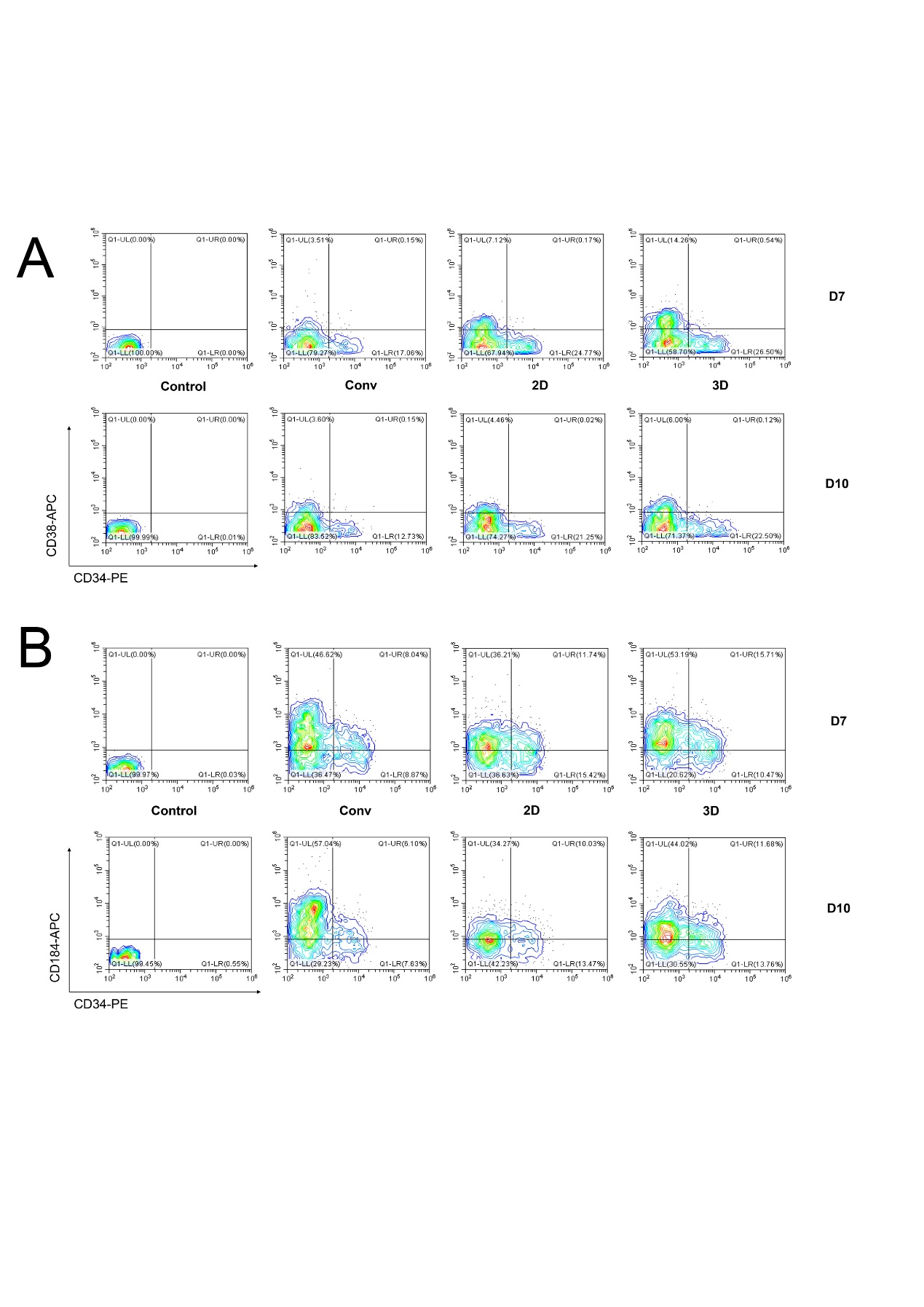


Supplementary Figure S1. Representative density plots of CD34+CD38-(A) and CD34+CD184+(B) phenotype of harvest cells on day 7 and day 10.
